# Synergistic bimetallic MOF/GO nanocomposite as a high-performance bifunctional electrocatalyst for the oxygen and hydrogen evolution reactions

**DOI:** 10.1039/d6ra01235c

**Published:** 2026-08-13

**Authors:** Muhammad Asghar, Ashi Rashid, Rashid Jalil, Badriah Mesfer Alotaibi, Munawar Iqbal, Abid Ali

**Affiliations:** a Department of Chemistry, University of Engineering and Technology Lahore Pakistan; b School of Chemistry and Mechanical Engineering, University of Leeds LS2 9JT UK; c Department of Physics, University of Engineering and Technology Lahore Pakistan; d Department of Physics, College of Science, Princess Nourah bint Abdulrahman University P.O. Box 84428 11671 Riyadh Saudi Arabia; e Zero Emission Technologies Innovation Center, University of Tabuk Tabuk 47913 Saudi Arabia; f Department of Chemistry, Faculty of Science, University of Tabuk Tabuk 47913 Saudi Arabia; g Department of Chemistry, The University of Lahore 1-Km Defence Road Lahore Pakistan abid.ali@chem.uol.edu.pk Bmalotaibi@pnu.edu.sa

## Abstract

The development of efficient, stable and non-precious bifunctional electrocatalysts for overall water splitting is crucial for sustainable energy solutions. This work presents the rational design and solvothermal synthesis of a bimetallic NiCu-MOF anchored on graphene oxide (GO) to form a synergistic NiCu-MOF@GO composite. Comprehensive characterizations *via* FTIR spectroscopy, XRD, SEM, EDX and UV-vis spectroscopy confirm the successful formation of the crystalline MOF structure and its uniform integration with the conductive GO network. When evaluated electrochemically, the NiCu-MOF@GO composite demonstrates exceptional activity for both the oxygen evolution reaction (OER) and hydrogen evolution reaction (HER) in an alkaline medium. It achieves significantly lower overpotentials and Tafel slopes than the pristine NiCu-MOF. Chronoamperometry and electrochemical impedance spectroscopy (EIS) further reveal outstanding long-term stability and a markedly reduced charge transfer resistance. The enhanced performance is attributed to the synergistic interplay, where the GO matrix prevents MOF aggregation, drastically improves electrical conductivity and increases the electrochemically active surface area, while the bimetallic NiCu system provides optimal active sites. This study underscores the immense potential of multivariate MOF/GO composites as high-performance, durable electrocatalysts for clean energy generation.

## Abstract 2

As an alternative to precious metal, NiCu-MOF@GO was fabricated through the integration of NiCu-MOF and GO, which effectively prevents MOF agglomeration, enhances charge transfer and improves bifunctional HER/OER performance and stability.

## Graphical abstract

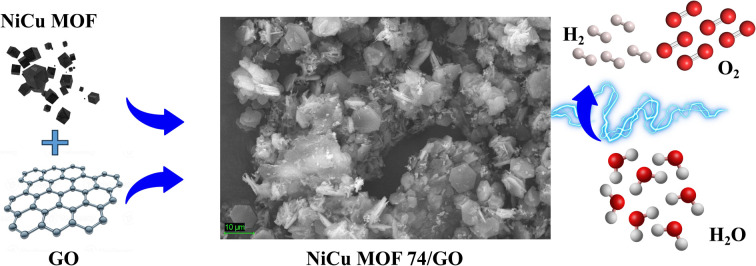

## 1 Introduction

The rapid expansion of the global population and industry has led to increased energy demand, overstressing infrastructure and exhausting natural resources.^1^ In today's world, 80% of the total energy used comes from fossil fuels, which increase greenhouse gas emissions and environmental degradation.^2^ Therefore, it is urgent to implement sustainable energy systems. Hydrogen can be considered a suitable choice because of high energy efficiency and lack of carbon dioxide emissions.^3^ However, large-scale hydrogen production is hindered by high costs and energy-intensive methods, driving research toward efficient alternatives like electrochemical water splitting. This process comprises the HER and the OER, with the latter being particularly sluggish due to its complex four-electron transfer process and high kinetic barriers.^4–6^ Although noble metals and their oxides, like Pt, IrO_2_ and RuO_2_, exhibit exceptional catalytic activity for these reactions, their high cost, scarcity and limited durability impede their widespread industrial adoption, underscoring the need for developing efficient, low-cost electrocatalysts.^7–9^

In order to overcome these constraints, there has been a recent trend in focusing on the synthesis of cheap and earth-abundant transition metals like Ni, Co, Fe, Cu and Mn as catalysts in place of noble metals.^10–16^ Recent studies have demonstrated that nanostructured materials and nanocomposites can dramatically improve catalytic performance due to their enlarged electrochemically active surface area, tunable morphology and abundant edge or defect sites that facilitate charge transfer. Despite these advantages, nanocomposites often suffer from particle agglomeration, unstable active sites and poor electronic conductivity, which limit their long-term catalytic stability and efficiency.^17,18^ In order to cope with these limitations, the use of metal organic frameworks (MOFs), which are crystalline and porous materials based on the coordination between metal ions and organic linkers, has gained much attention as the new generation platform for designing advanced electrocatalysts.^19,20^ Their high surface area, ordered porosity and structural tunability make MOFs excellent candidates for constructing advanced catalysts with accessible active sites and tailored electronic properties.^21–23^

The bimetallic MOFs are highly efficient catalysts for electrochemical water splitting owing to the combined effects of two metal centers leading to synergistic electronic tuning, redox reactivity and adsorption behavior of intermediates.^24^ For instance, the NiFe-MOF,^25^ CoFe-MOF,^26^ NiCo-MOF,^27^ and CuCo-MOF^28^ have demonstrated enhanced OER/HER performance compared to their monometallic counterparts due to multiple accessible oxidation states and abundant catalytic sites. However, pristine MOFs usually suffer from poor electrical conductivity and instability while undergoing the electrolysis process.^20^ To counter such problems, carbonaceous materials such as graphene have been used owing to high stability and electrical conductivity. Unfortunately, it is difficult to functionalize the material and it is also hydrophobic in nature.^29^ Conversely, graphene oxide has been widely employed as a conductive and mechanically robust support.^30^ Its high electrical conductivity, large surface area, oxygen-containing functional groups and excellent chemical stability facilitate fast electron transport, strong MOF anchoring and suppression of particle agglomeration, ultimately enhancing the catalytic activity and durability of MOF-based composites for efficient overall water splitting.^31,32^

The synthesized bimetallic framework is named NiCu-MOF-74, which is a member of the well-known MOF-74 family (also referred to as CPO-27). Its nomenclature is based on the coordination of divalent metal nodes (Ni^2+^ and Cu^2+^) to an organic linker (2,5-dihydroxyterephthalic acid) to produce characteristic one-dimensional hexagonal porous channels. The unique and synergistic characteristics of the NiCu-MOF-74@GO heterostructure, as reported in this work, render it a system with a distinct and outstanding water splitting performance, compared to the other reported systems. The novelty of this catalyst lies in a three-fold structural and electronic engineering approach. The first is the synergistic bimetallic effect between Ni and Cu atoms in the framework, which promotes the rational electronic modulation of the catalyst, leading to an increase in the binding energies of the catalytic intermediates and the intrinsic bifunctional activity. Second, the unique topology of the MOF-74 structure opens large 1D hexagonal channels that show an extraordinary density of coordinatively unsaturated open metal sites in the direct vicinity of the electrolyte. Finally, this bimetallic framework can be directly nucleated on a 2D graphene oxide (GO) scaffold to address the intrinsic low electrical conductivity of bulk MOFs. This excellent interfacial coupling between the MOF nodes and the oxygen functional groups of GO not only provides fast electron transfer pathways, but also avoids MOF agglomeration and structural collapse during continuous gas evolution. Therefore, this engineered composite is a very efficient and strong bifunctional electrocatalyst.

This is where the recent advances in the field of electronic modulation and structural hybridization of bimetallic structures come in. The local electronic environment and d-band center of the active sites can be modified in a strategic way by introducing a secondary transition metal (*e.g.*, Cu) into the Ni-based MOF, which is not based on any scaling principles and optimizes intermediate binding energies. In addition, a 2D conductive matrix, such as graphene oxide (GO), can be used for the hybridization of the MOF to create a continuous long-range electron transport pathway, which would overcome the conductivity issue. Hence, it is extremely important to study the synergistic effects of bimetallic NiCu nodes confined in a GO scaffold for the development of the next generation of efficient bifunctional MOF-based electrocatalysts.

To cover all these knowledge gaps, a bimetallic NiCu-MOF and its graphene oxide composite (NiCu-MOF@GO) were successfully synthesized *via* a facile solvothermal method. Comprehensive characterizations using FTIR and UV-vis spectroscopy, XRD, SEM and EDX unequivocally confirmed the successful formation of the MOF structure, the integration of both metal nodes and the homogeneous distribution of the MOF on the conductive graphene oxide sheets. The subsequent electrochemical evaluation revealed the superior bifunctional performance of the NiCu-MOF@GO composite. It demonstrated exceptional activity and stability for both the OER and HER. As validated by electrochemical analysis, it enhanced charge transfer kinetics. This study not only underscores the synergistic advantage of the bimetallic NiCu system within the MOF-74 architecture but also highlights the critical role of graphene oxide in boosting electrical conductivity and active site accessibility. The NiCu-MOF@GO composite emerges as a highly promising and durable nonprecious electrocatalyst for overall water splitting, presenting a significant stride towards efficient clean energy conversion.

## 2 Materials and methods

### 2.1 Materials

Nickel(ii) nitrate hexahydrate (Ni(NO_3_)_2_·6H_2_O, 99.9%) and copper(ii) nitrate trihydrate (Cu(NO_3_)_2_·3H_2_O, 99.9%) were obtained from Alfa Aesar and the organic linker 2,5-dihydroxyterephthalic acid (DHTA, 98%) was obtained from TCI Chemicals. *N*,*N*-Dimethylformamide (DMF, 99.8%, Sigma-Aldrich), absolute ethanol (99.9%, Merck) and deionized water were used. For the synthesis of graphene oxide *via* the Hummers' method, a natural graphite powder (99.99%, Sigma-Aldrich), sodium nitrate (NaNO_3_, 99%, Sigma-Aldrich), potassium permanganate (KMnO_4_, 99%, Sigma-Aldrich), concentrated sulfuric acid (H_2_SO_4_, 98%, Sigma-Aldrich) and hydrogen peroxide (H_2_O_2_, 30% w/w, Sigma-Aldrich) were employed. The purification process involved hydrochloric acid (HCl, 37%, Sigma-Aldrich) and deionized water. Finally, potassium hydroxide (KOH, 85%, Sigma-Aldrich) was used to prepare the electrolyte for electrochemical measurements. All chemicals and reagents were of analytical grade and used as received without further purification.

### 2.2 Synthesis of NiCu-MOF-74

The bimetallic NiCu-MOF with an equimolar metal ratio was synthesized *via* a solvothermal method. In a typical procedure, 0.5 mmol of DHTA and a total of 1.5 mmol of metal precursors, specifically 0.75 mmol of Ni(NO_3_)_2_·6H_2_O and 0.75 mmol of Cu(NO_3_)_2_·3H_2_O, were dissolved in a mixed solvent system containing 15 mL of DMF, 1 mL of deionized water and 1 mL of ethanol. The resulting solution was transferred to a 20 mL Teflon-lined stainless-steel autoclave and heated at 105 °C for 24 hours. After cooling to room temperature, the solid product was collected by centrifugation at 10 000 rpm for 3 minutes. The product was then washed thoroughly with DMF and methanol several times to remove unreacted precursors and impurities. Finally, the purified product was dried at 80 °C under dynamic vacuum for 12 hours. The obtained sample was then stored at 4 °C for further use.^33^

### 2.3 Synthesis of graphene oxide

Graphene oxide (GO) was synthesized from a natural graphite powder using the modified Hummers' method. In a typical procedure, 1 g of graphite and 0.5 g of sodium nitrate (NaNO_3_) were added to 23 mL of concentrated sulfuric acid (H_2_SO_4_, 98%) in an ice bath (0 °C-5 °C) under constant stirring. After 1 hour, 3 g of KMnO_4_ was added to the suspension gradually, ensuring that the reaction temperature did not exceed 20 °C to avoid overheating and an explosion. After the removal of the ice bath, the temperature of the mixture was increased to 35 °C, where stirring continued for 12 hours, yielding a paste-like substance. In the next step, 46 mL of deionized water was added to the paste; this resulted in an exothermic reaction and a temperature rise to 98 °C. The reaction was then terminated by the addition of 140 mL of deionized water, followed by the addition of 10 mL of hydrogen peroxide (H_2_O_2_, 30%), which reduced the residual permanganate and manganese dioxide to soluble sulfate salts, turning the color of the suspension from dark brown to brilliant yellow. The resulting product was repeatedly washed and centrifuged with a 5% hydrochloric acid (HCl) solution and then with deionized water until the supernatant reached a neutral pH. The final graphene oxide slurry was collected and dried in a vacuum oven at 60 °C for 24 hours to obtain a solid. This solid was then exfoliated by mild sonication in water to create a stable graphene oxide dispersion for further use.^16^

### 2.4 Synthesis of NiCu-MOF@GO composites

The NiCu-MOF@GO composite was synthesized *via* a one-pot solvothermal method. First, 50 mg of pre-synthesized graphene oxide was dispersed in a mixed solvent system containing 15 mL of DMF, 1 mL of deionized water and 1 mL of ethanol *via* ultrasonication for 30 minutes to achieve a homogeneous dispersion. To this well-dispersed GO solution, 0.5 mmol of 2,5-dihydroxyterephthalic acid (DHTA) and a total of 1.5 mmol of metal precursors, specifically 0.75 mmol of Ni(NO_3_)_2_·6H_2_O and 0.75 mmol of Cu(NO_3_)_2_·3H_2_O, were added sequentially under vigorous stirring. The resulting mixture was stirred for an additional hour to ensure the thorough mixing of all components. The suspension was then transferred to a 20 mL Teflon-lined stainless-steel autoclave and heated at 105 °C for 24 hours. After the autoclave cooled naturally to room temperature, the solid product was collected by centrifugation at 10 000 rpm for 3 minutes. The recovered composite was washed thoroughly with DMF and methanol several times to remove unreacted species and impurities. Finally, the purified NiCu-MOF@GO composite was dried at 80 °C under dynamic vacuum for 12 hours and stored at 4 °C for subsequent use.

### 2.5 Electrochemical analysis

A Gamry ref. 6000 potentiostat/galvanostat was used to measure all OER activities using a three-electrode system. A NiCu-MOF@GO-modified CC was used as the working electrode and Ag/AgCl and a platinum sheet were used as the reference and counter electrodes, respectively. A 1 M KOH aqueous solution was used as the electrolyte. The linear sweep voltammetric (LSV) curves were recorded in the redox potential range of 0–1 V (*vs.* Ag/AgCl reference electrode) at a scan rate of 5 mV s^−1^. This potential was then converted to the reversible hydrogen electrode (RHE) value using eqn (1) as follows:

1 *E*_RHE_ = *E*_Ag/AgCl_ + *E*_0_ + (0.059 × pH)

The LSV data were used to plot the Tafel slope and to study reaction kinetics. To investigate the linear Tafel slope, eqn (2) was used as follows:2
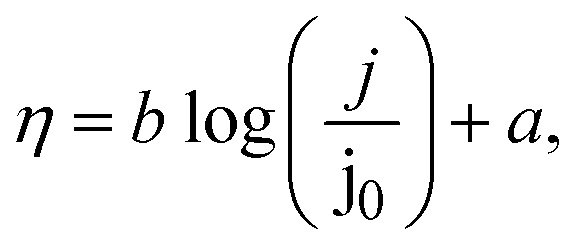
where *η* is the overpotential, *j*_0_ is the exchange current density, *j* is the current density, *b* is the Tafel slope and ‘*a*’ is a constant related to the activation energy of the reaction. The double-layer capacitance (*C*_dl_) of the electrode material was calculated using CV curves. The electrochemically active surface area (ECSA) was determined from the value of *C*_dl_. Impedance measurements were performed using a 0.1 M KOH aqueous electrolyte solution through the use of electrochemical impedance spectroscopy (EIS). The frequency range was determined to be from 0.1 Hz to 1 MHz when the voltage of the OER and HER was 0.65 V.

The polarization curves of all linear sweep voltammetry (LSV) experiments and the reported overpotentials were *iR*-corrected to remove the ohmic voltage drop (due to the electrolyte resistance). The uncompensated solution resistance (*R*_s_) for every measurement was obtained from the respective electrochemical impedance spectroscopy (EIS) Nyquist plots. The potentials were corrected from the measured potentials using the equation *E*_corrected_ = E_measured_ − I × *R*_s_, for manual (or automatic) correction by the potentiostat, to get the real intrinsic catalytic activities of the fabricated electrodes.

### 2.6 Characterization

To accurately evaluate the crystallographic structure, morphology and physicochemical properties of the synthesized materials, comprehensive characterizations were performed under strictly controlled conditions.

X-ray diffraction (XRD, Bruker D8 Advance) with Cu Kα radiation (*λ* = 1.5406 Å) was used to identify the crystalline phases of the samples. The measurements were taken from 10° to 80° (2*θ*) with a step size of 2° min^−1^ at a 40 kV applied voltage and 30 mA applied current. All powder samples were finely ground in an agate mortar before XRD to achieve a uniform particle size. Fourier transform infrared (FTIR) spectroscopy (PerkinElmer) was used to perform functional group analysis. The solid samples were ground to very fine powders, mixed with KBr (spectral grade) in a mass ratio of about 1 : 100 and pressed into translucent pellets, whose spectra were obtained in the range of 4000–400 cm^−1^. Surface morphologies and elemental distributions were investigated by scanning electron microscopy (SEM, Hitachi) equipped with energy-dispersive X-ray spectroscopy (EDS). The sample powders were mounted on conductive carbon tape and a thin layer of gold (or carbon) was sputtered onto the powders to prevent charging and to enhance the image resolution. The measurements were taken at a high voltage of 15 kV. The UV-Vis absorption spectra were recorded using a UV-Vis spectrophotometer (Shimadzu). To achieve transparency, sample preparation was carefully controlled for the NiCu-MOF@GO powders, as they were opaque and scattered light. Specifically, 1 mg of the sample was introduced into 10 mL of deionized water to obtain a highly diluted sample suspension (0.1 mg mL^−1^). This mixture was continuously ultrasonicated for 30 minutes to obtain a stable, uniform colloidal dispersion and immediately analysed in a standard 1 cm quartz cuvette to minimise light-scattering artifacts.

## 3 Results and discussion

### 3.1 SEM micrographs

**Fig. 1:**
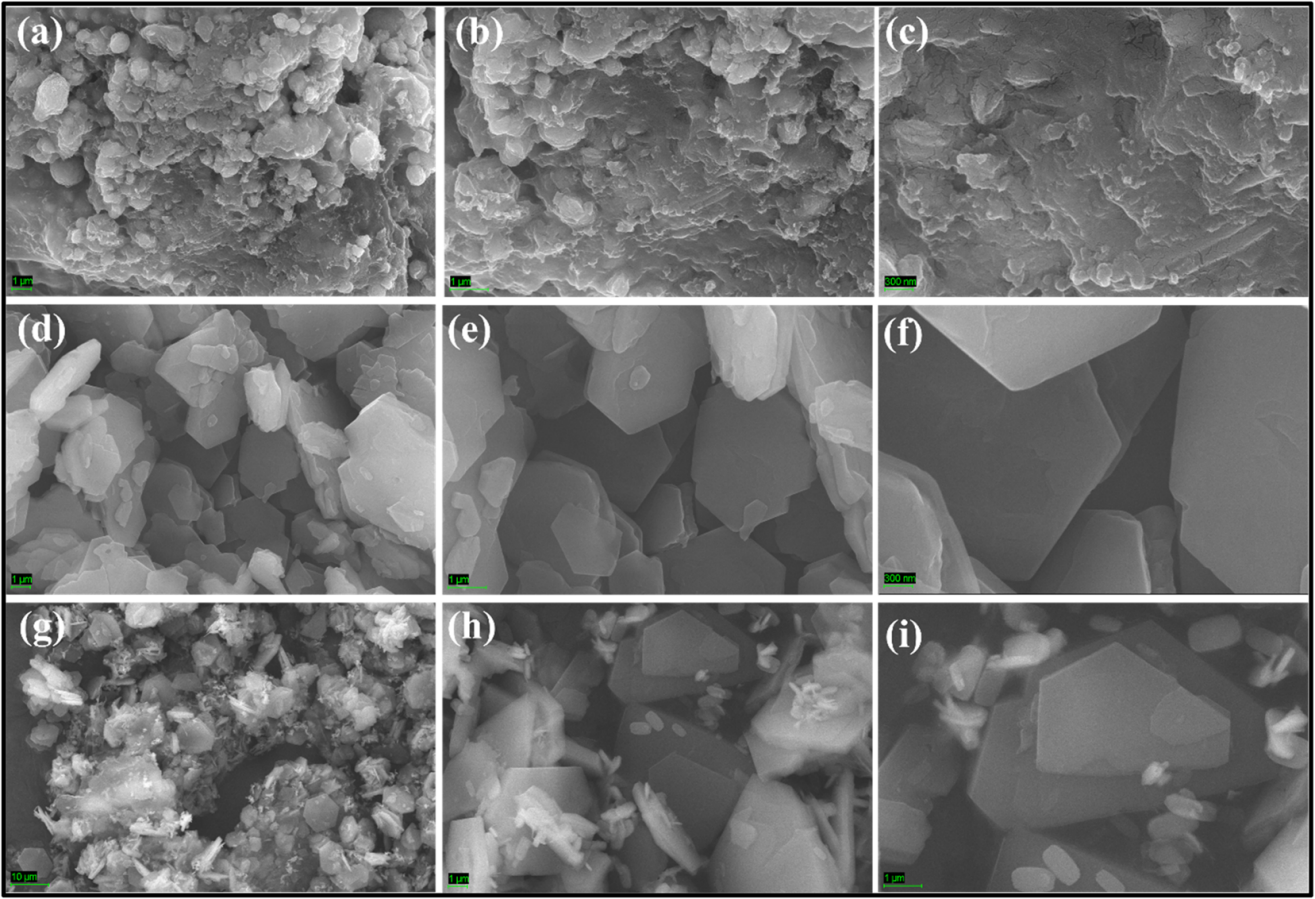
SEM micrographs of GO (a–c), NiCu-MOF-74 (d–f) and the NiCu-MOF@GO composite (g–i) at different resolutions.

The surface morphology of the synthesized materials was studied by SEM. The surface morphology of pristine GO produced using the modified Hummers' method is shown in Fig. 1a–c. The GO is not as thin as 2D sheets or a single layer, but rather is thick and highly wrinkled with agglomerated multilayer structures. This “bulk powder drying” process-induced aggregated morphology is well documented. The dense, crumpled structure is a result of the strong inter-layer hydrogen bonding and van der Waals attraction forces between the highly oxygenated graphene sheets when the solvent is removed.

The micrographs of the pristine NiCu-MOF-74 clearly show that it has hexagonal and polygonal flat flakes as opposed to a rod-like morphology (Fig. 1d–f). The NiCu-MOF@GO composite (Fig. 1g–i) consists of flat MOF flakes that are tightly embedded in the GO matrix. Notably, the typical, free-standing, thin-layered structure that is often associated with pristine graphene oxide is not clearly visible in the composite's images. On the contrary, the morphology is found to be aggregated graphene basal planes interlocked with flakes of the MOF. This morphology is due to the synergistic effect that occurs between the GO scaffold and the growing MOF crystals in the synthesis process.^33^

The *in situ* nucleation of the NiCu-MOF is quite strong and the main anchoring site is the abundant oxygen-rich functional groups on the GO surface. Thus, a dense crystal proliferation takes place directly across the basal planes, camouflaging the underlying 2D graphene sheets. Furthermore, the amount of partial reduction in the GO sheets is expected to be high during the hydrothermal/solvothermal process. There are strong interplanar π–π stacking interactions with van der Waals forces that tend to cause the re-stacking and agglomeration of the graphene basal planes. Thus, in Fig. 1g–i, the resulting structure has a very high degree of integration, with a dense population of flakes of the MOF and aggregated, restacked GO planes forming a structured matrix, from which the dispersion of individual 2D layers of graphene cannot be observed.^34^

### 3.2 FTIR analysis

**Fig. 2:**
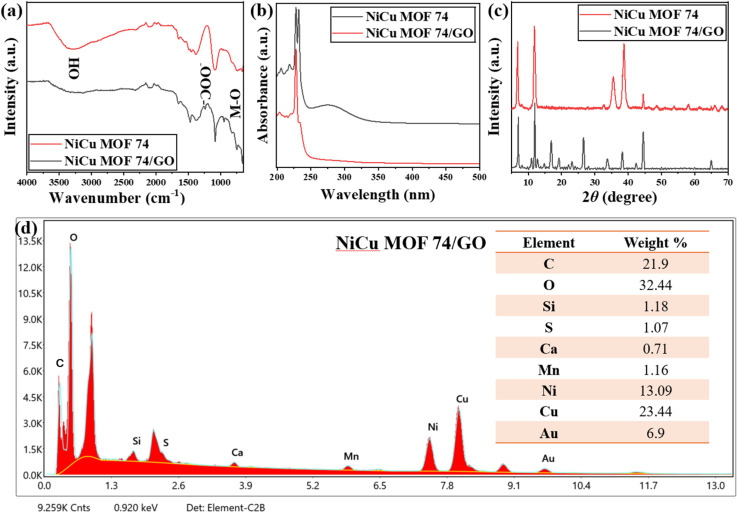
Comprehensive material characterization of the synthesized GO, NiCu-MOF and NiCu-MOF@GO composite. (a) FTIR spectra confirming the functional groups and metal-linker coordination. (b) UV-vis spectra showing the optical absorption and electronic properties. (c) X-ray diffraction (XRD) patterns verifying the crystalline phase and structure. (d) EDX spectrum of the NiCu-MOF@GO composite with the elemental weight (%) composition table.

The Fourier transform infrared (FTIR) spectroscopic analysis provides definitive evidence for the successful synthesis of the NiCu-MOF and its subsequent integration with graphene oxide, as shown in Fig. 2a. The spectrum of the pristine NiCu-MOF, utilizing DHTA as the organic linker, confirms deprotonation and coordination through the absence of a characteristic free carboxylic acid peak (∼1700 cm^−1^) and the presence of asymmetric and symmetric carboxylate stretching vibrations at 1647 cm^−1^ and 1379 cm^−1^, respectively.^35^ The separation (Δ*ν*) of approximately 268 cm^−1^ between these peaks indicates a bridging coordination mode of the carboxylate groups to the metal clusters (Ni^2+^/Cu^2+^), forming the foundational secondary building units of the MOF.^36^ Furthermore, the broad band centered at 3300 cm^−1^ signifies O–H stretching from the linker hydroxyl groups and adsorbed water^37^ involved in extensive hydrogen bonding, while the peaks in the fingerprint region, such as those at 738 cm^−1^ and 663 cm^−1^, are assigned to aromatic C–H bending and metal–oxygen (M–O) vibrational modes, respectively, completing the structural fingerprint.^38^

The formation of NiCu-MOF@GO composite is evaluated by the spectral comparison. While the main MOF structure is preserved, the significant changes in the frequencies of vibrations, such as the shifting of the asymmetric carboxylate stretching band from 1647 cm^−1^ to 1654 cm^−1^ indicate changes in the chemical environment of the metal clusters, which can be a result of the interaction between the graphene oxide and the composite. The strongest evidence of the success of the synthesis procedure is provided by the appearance of the new distinct peaks in the spectrum at 1237 cm^−1^ and 946 cm^−1^, which unambiguously indicate the presence of the C–O–C epoxy group and other oxygen functionalities characteristic of the graphene oxide structure.^39^ This, coupled with the changes in the M–O region, confirms that the integration extends beyond physical mixing to include supramolecular interactions such as π–π stacking and hydrogen bonding, which are anticipated to synergistically enhance the composite's structural and electronic properties.

### 3.3 UV-vis spectroscopic analysis

The UV-visible spectroscopy is employed for the optical and electronic characteristics evaluation. The NiCu-MOF possesses a pronounced absorption edge in the ultraviolet range due to the ligand-to-metal charge transfer (LMCT) from the oxygen atom of the DHTA linker to the metal atoms (Ni^2+^ and Cu^2+^), as illustrated in Fig. 2b. An absorption peak associated with the d–d transitions of the Cu^2+^ ions may be present. In the case of the NiCu-MOF@GO composite, a marked redshift (or bathochromic shift) of this absorption edge toward higher energy can be detected.^40^ This shift signifies a reduction in the effective band gap of the material, which is a direct consequence of the strong electronic coupling between the MOF and graphene oxide.^41^ The conductive graphene oxide network introduces new electronic states, facilitating easier charge transfer and enhancing the material's light-harvesting capability. This synergistic effect is crucial for improving the efficiency of electrochemical processes by promoting better charge separation and migration.

### 3.4 XRD analysis

The X-ray diffraction (XRD) patterns are fundamental for confirming the crystalline phase and structural integrity of the synthesized materials. The pristine NiCu-MOF sample should exhibit a diffraction pattern that closely matches the simulated pattern of the iso-structural MOF-74 framework. The MOF-74 structure is known for its characteristic honeycomb topology, which produces several distinct low-angle peaks, as shown in Fig. 2c. The most intense peak is typically observed at 2*θ* = 5.9°, corresponding to the (110) crystallographic plane, which reflects the periodicity of the one-dimensional hexagonal channels. The other significant peak at 2*θ* = 10.9° can be indexed to the (200) plane, confirming the long-range order and high crystallinity of the bimetallic framework.^33^ The appearance increased higher angle reflections, especially at 2*θ* = 40° and further, which correspond to the (221) and (401) planes, proves long-range atomic order and high crystallinity of the bimetallic framework over the lattice. Lack of additional peaks from nickel and copper oxide phases shows the formation of a pure bimetallic solid solution.

The XRD pattern of the NiCu-MOF@GO composite is of particular importance in understanding the successful integration. First and foremost, the fact that all main characteristic peaks of the NiCu-MOF are preserved can be seen. This means that the crystalline structure of the MOF remains stable throughout the process of composite synthesis and the presence of graphene oxide does not lead to its destruction. The difference between the pattern of the composite and that of the pure material, therefore, lies in the presence of a very wide, low-intensity hump at 2*θ* = 10–15°, which is due to the (001) peak of disordered exfoliated graphene oxide sheets. Since the sharp peaks of MOF prevail in the pattern, while the signal from the graphene oxide is very weak and broad, this implies that MOF crystals are the main crystalline phase of the composite and GO sheets are separated and decorated with MOF particles without being able to re-stack together.

The diffraction pattern shows that there is a synergistic interaction between the NiCu-MOF and the GO scaffold. The NiCu-MOF@GO composite has slightly sharper diffraction peaks and a slightly lower relative crystallinity compared to the pristine bulk MOF. This modification suggests that the 2D GO sheets successfully confine the nucleation and growth of the MOF to smaller nano-sized crystallites, which are extremely desirable for exposing a higher density of catalytically active sites. In addition, minute changes in the peak positions are seen in the XRD pattern of the composite compared to that of the pure MOF. These changes correspond to lattice strain due to strong coupling at the interfaces. Direct chemical coordination bonding (M–O–C) can be formed between the metal (Ni and Cu) nodes of the framework and the oxygen-rich functional groups on the surface of GO, which can serve as heterogeneous nucleation sites. The chemical anchoring mechanism not only prevents the aggregation of MOF particles and the restacking of GO sheets but also creates an electronic synergy at the interface to enable fast charge transfer between components, which is essential for efficient electrocatalysis.

### 3.5 EDX analysis

Quantitative elemental confirmation can be achieved through the EDX analysis. In the case of NiCu-MOF, the elements Ni and Cu were observed along with carbon and oxygen from the organic ligand that dominates the spectrum. In the case of the NiCu-MOF@GO composite, the carbon peak becomes significantly more intense as an indication of successful addition of GO rich in carbon content. Elemental mapping, where Ni (green), Cu (blue) and C (red) elements should be seen uniformly in the same particle agglomerate, would be an indication that bimetallic MOFs are successfully mixed together with the GO substrate.

### 3.6 Electrochemical activity

**Fig. 3:**
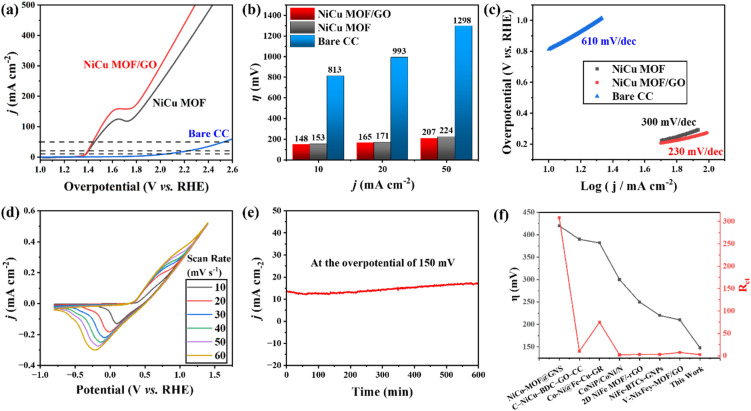
(a) Linear sweep voltammetry (LSV) curves. (b) Bar chart comparing the overpotentials (*η*) at a specific current density. (c) Corresponding Tafel plots derived from the LSV data. (d) CV cycles at different scan rates (for the faradaic region). (e) Chronoamperometry curve. (f) Comparison of the overpotential (*η*) and charge transfer resistance (*R*_ct_) of the synthesized composite material and recently reported MOF-graphene composites.

From the electrochemical data shown in Fig. 3a–f, it is evident that the NiCu-MOF@GO composite material exhibits better OER activity owing to the synergistic effect between the two-component structure.

#### 3.6.1 Oxygen evolution reaction

Linear sweep voltammetry depicted in Fig. 3a represents a measure of the OER efficiency. Indeed, the composite demonstrates a distinct leftward shift in comparison with its constituent elements and various other catalysts, which means that less overpotential is needed to activate the OER reaction. This enhanced activity is quantified in Fig. 3b, where the overpotential (*η*) at a benchmark current density (*e.g.*, 10 mA cm^−2^) for the NiCu-MOF@GO composite is markedly lower (148 mV) than that of the pristine NiCu-MOF (165 mV) and other controls. This reduced overpotential is a direct metric of a more efficient and powerful electrocatalyst. Overpotential itself represents an important indicator of the efficiency of the electrocatalyst. OER reaction kinetics are evaluated with the help of the Tafel plots shown in Fig. 3c, which have been calculated using the data of the LSV analysis. The Tafel slope (230 mV dec^−1^) of the NiCu-MOF@GO sample is the lowest in comparison with all other catalysts, which means that the kinetics of the reaction are more favorable in terms of the catalyst's properties.

Improved performance originates from the intrinsic properties of the material. The CV cycles recorded at various scan rates (Fig. 3d) have been used to measure the ECSA based on double-layer capacitance (*C*_dl_). An increased *C*_dl_ in the composite indicates more active sites available; this is the result of the role played by graphene oxide in inhibiting the aggregation of MOF nanoparticles. In addition, EIS results from Fig. 3f demonstrate that the composite exhibits the lowest *R*_ct_. This proves that the graphene oxide matrix is capable of providing the best conductive path, thereby significantly increasing the speed of electrons moving from the active NiCu sites to the electrode surface. Durability is a key element that determines whether the electrocatalyst is practical for real-world applications. The chronoamperometry test in Fig. 3e shows a stable current density over a prolonged period (*e.g.*, 10 hours), confirming the robust structural and chemical stability of the NiCu-MOF@GO composite under harsh oxidative conditions. This stability is crucial for real-world applications. Finally, Fig. 3f provides a critical comparative analysis, benchmarking the composite's overpotential and *R*_ct_ against recently reported MOF-graphene composites. The fact that our NiCu-MOF@GO composite demonstrates competitive, if not superior, performance highlights its significance. There is synergy in the bimetallic system of NiCu that facilitates the adsorption of oxygenated intermediates, whereas the graphene oxide framework ensures maximum exposure of the active sites and also conductivity. This integrated approach results in a high-performance, stable and nonprecious OER electrocatalyst that stands favorably against contemporary research.

#### 3.6.2 Hydrogen evolution reaction (HER)

**Fig. 4:**
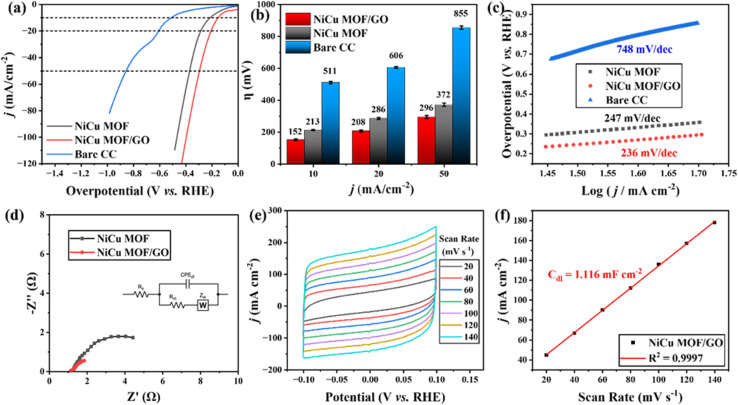
(a) Linear sweep voltammetry (LSV) curves. (b) Bar chart comparing the overpotentials (*η*) at a specific current density. (c) Corresponding Tafel plots derived from the LSV. (d) Electrochemical impedance spectra of the pristine NiCu-MOF and NiCu-MOF@GO composite. (e) CV cycles in the scan rate range from 20 mV s^−1^ to 140 mV s^−1^. (f) Double-layer capacitance (*C*_dl_) calculated from the CV cycles.

The electrochemical data presented in Fig. 4a–f provide a compelling and multi-faceted argument for the superior HER performance of the NiCu-MOF@GO composite, establishing its priority as a highly efficient non-precious metal electrocatalyst. The synergy between the bimetallic MOF and the conductive graphene oxide scaffold is the fundamental driver of this enhanced activity, which is confirmed through the metrics of activity, kinetics, conductivity and active site availability.

The primary evidence for the enhanced catalytic activity is derived from the LSV curves in Fig. 4a. The composite's curve demonstrates a pronounced positive shift, initiating at a more positive potential and achieving a significantly higher current density at a lower overpotential compared to the pristine NiCu-MOF and other benchmarks. This indicates that the composite requires a substantially smaller energy input to generate hydrogen gas at the same rate, a direct measure of a more efficient catalyst. Polarization curves of LSV were recorded to assess the HER activity, as shown in Fig. 4a. It is interesting to note that the NiCu-MOF@GO composite also has a non-zero cathodic current around 0 V *vs.* RHE before the rapid faradaic onset of the HER. This early baseline current is not due to hydrogen evolution, but rather results from the high capacitance of the electrical double-layer (*C*_dl_) created by the highly porous MOF and the high-surface-area GO scaffold. This is a very large solid/liquid interface and during the continuous voltage sweep, a clear non-faradaic background current is observed. Moreover, there is a small baseline pseudocapacitive effect resulting from the reduction of surface oxygen functional groups on the GO and the pre-catalytic redox transition of surface metal nodes (Ni/Cu). This capacitive region is followed by a faradaic HER current, which abruptly rises. This qualitative observation is quantitatively confirmed in Fig. 4b, where the overpotential (*η* = 152 mV) at a standard current density of 10 mA cm^−2^ for the composite is drastically lower. A lower overpotential is a critical performance metric, directly translating to higher energy efficiency for hydrogen production and it positions the composite favorably against many reported non-precious catalysts.

Beyond mere activity, the reaction kinetics, which dictate how efficiently the current density increases with the applied potential, are superior for the composite. The Tafel plots in Fig. 4c, derived from the LSV data, show a significantly lower Tafel slope of 236 mV dec^−1^ for the NiCu-MOF@GO composite compared to the pristine NiCu-MOF (247 mV dec^−1^). Lower values of Tafel slope are indicative of better kinetics, implying that either the Volmer–Heyrovsky or Volmer–Tafel mechanism takes place at lower energy barriers. Better kinetics mean that not only will the process take place easily, but also the rate of the process will be accelerated without any further need for higher voltage.

The Tafel slopes for both the HER (236 mV dec^−1^) and OER are still relatively high, but the integration of GO has greatly enhanced the kinetic parameters compared with the pure MOF. This Tafel slope implies that the first dissociation of water (the Volmer step, H_2_O + e^−^ → H_ads_ + OH^−^) is the main rate-determining step and a high kinetic barrier for intermediate adsorption at the catalytic sites suggests that the Volmer step is significant. In addition, the intrinsic structural limitations of the MOF are a cause of the sluggishness. The MOF-74 structure has uniform 1D micropores, which offer a large number of active sites; however, the narrow channels may limit mass transport when strong gas evolution occurs. The formation of bubbles of H_2_ and O_2_ gases inside or on the micropores probably prevents the electrolyte from reaching the active sites, causing an increase in the local resistance and a sharper Tafel slope.

It is possible that these kinetic limitations can be overcome through future optimizations of the structure. Some possible approaches are the introduction of hierarchical porosity (adding mesopores to allow for quick desorption of gas bubbles), targeted defect engineering (optimizing the number of open metal sites), and the *in situ* growth of the composite on 3D conductive substrates without the need for polymeric binders such as Nafion, which would reduce the interfacial charge transfer resistance further.

The origin of this exceptional activity and kinetics is rooted in the improved electronic structure and charge transport properties conferred by the graphene oxide matrix. The electrochemical impedance spectroscopy (EIS) Nyquist plots in Fig. 4d offer direct evidence of this. The composite shows a significantly smaller diameter for the semicircle than that of the pristine MOF. This translates into a significantly smaller value of charge transfer resistance (*R*_ct_). This reduction in *R*_ct_ clearly demonstrates the presence of highly effective pathways for electrons to travel from the electrode to the MOF sites. This rapid electron transport is essential for the multi-step HER process, preventing kinetic bottlenecks and ensuring that protons are reduced to hydrogen gas with maximum efficiency.

Moreover, the improved performance of the composite is achieved owing to the increased availability of catalytic sites. In the case of the CV scans conducted at different scan rates (20 to 140 mV s^−1^), depicted in Fig. 4e, the non-faradaic capacitive current for the composite is more pronounced and constant. This is because of the charging of the double layer and not because of any redox reaction. From the graph plotted in Fig. 4f between the charge current density and scan rate, the *C*_dl_ of the composite can be calculated. The increased value of the *C*_dl_ is directly related to the increase in ECSA. Thus, it is evident that the presence of GO sheets is responsible for restricting the agglomeration of NiCu-MOF nanoparticles and thereby increasing the available number of catalytic sites to a significant extent. This is the key reason behind the high performance of the composite. The synergistic effect is manifested in quantitative terms such as reduced overpotential, fast reaction rate kinetics (reduced Tafel slope), greatly enhanced charge transfer (decreased *R*_ct_) and increased number of active sites (increased *C*_dl_). The combination of these parameters, which occurs in this composite, results in an electrocatalyst that is both highly active and efficient as well as highly stable. The overall improvement in performance of this kind makes the NiCu-MOF@GO composite a highly competitive candidate for hydrogen production.

The synergistic effect of the NiCu-MOF with GO combination surpasses the performance potential of the individual constituents, which enhances the efficiency of the composite material. The interaction between the components is based on their structure and electronic nature: the two-dimensional GO layers serve as a conducting support for the MOF, preventing their aggregation and thus providing access to an abundant number of metallic active sites, which can be confirmed by SEM analysis and increased *C*_dl_. At the same time, this interaction results in a highly efficient electronic connection, resulting in the reduction of the charge transfer resistance and rapid electron transfer to the catalyst active sites. This makes the process of intermediate adsorption more efficient and the reaction kinetics faster, which leads to the extremely low overpotential and Tafel slopes for both OER and HER processes. Consequently, the unique combination of the chemically modified MOF and the excellent conductivity of GO leads to the formation of an outstanding bifunctional electrocatalyst.

The unprecedented bifunctional catalytic activity of the NiCu-MOF@GO composite is believed to be driven by a synergistic mechanism that involves the combination of both bimetallic redox couples and the carbonaceous scaffold. The lattice incorporation of Cu and Ni at the atomic level leads to strong electronic modulation. This bimetallic synergy results in the modification of the local charge distribution and maximizes the d-band centre of the active sites. This allows for fine-tuning of the chemisorption energies of critical reaction intermediates (*e.g.*, OOH˙, OH˙ and O˙ for the OER and H˙ for the HER), which is not usually possible in monometallic catalysts. Besides, the Cu nodes synergistically reduce the activation energy for the structural transformation of the surface Ni species to highly active NiOOH, the key catalytic center in the process of the alkaline OER.

At the same time, the charge-transfer kinetics are greatly dependent on the 2D graphene oxide (GO) matrix. The GO sheets offer a highly conductive network of interconnected sheets, which would otherwise have been lacking in pristine MOFs. The fast charge transfer between the MOF nodes and the surface of GO helps to reduce the charge transfer resistance at the GO-catalyst interface, which leads to rapid electron shuttle from the catalytic metal centers to the current collector. The combination of Ni/Cu electronic modulation with the GO matrix affords highly active local sites and the rapid macroscopic charge transfer ensures high current electrolysis.

This enhanced electrocatalytic activity of the NiCu-MOF@GO composite can be attributed to the highly synergistic effect of NiCu metals and the conductive GO scaffold. MOFs of Ni often have high oxygenated intermediate binding energies, but by incorporating Cu, the local electronic environment is modulated. The atomic substitution of Ni by Cu results in lattice strain from the point of view of the structure since the diffraction planes in the XRD shift. This lattice distortion changes the d-band center of the bimetallic active sites.

From a mechanistic perspective, the OER pathway in alkaline media proceeds *via* four coupled electron-proton transfer steps:M + OH^−^ → M–OH + e^−^, M–OH + OH^−^ → M–O + H_2_O + e^−^, M–O + OH^−^ → M–OOH + e^−^, M–OOH + OH^−^ → M + O_2_ + H_2_O +e^−^,where M represents the active metal site.

The high thermodynamic energy barrier for the conversion of the ˙O intermediate to the ˙OOH intermediate is usually the rate-limiting step (RDS) in monometallic Ni systems. The addition of Cu is an electronic modulator. Because of the electronegativity difference between Ni (1.91) and Cu (1.90), there is an internal charge transfer, which optimises the filling of the e_g orbitals of the Ni active centres. This electronic reconfiguration reduces the overly strong adsorption of *O and stabilises the formation of the intermediate, decreasing the overall overpotential.

### 3.7 Synergism of the MOF and graphene

The integration of the NiCu-MOF with graphene oxide orchestrates a powerful synergy that transcends the simple sum of its components, catapulting the composite's electrocatalytic performance to new heights. This partnership is fundamentally structural and electronic: the two-dimensional graphene oxide sheets act as a conductive scaffold, effectively suppressing the aggregation of MOF nanorods and exposing a vast landscape of accessible bimetallic active sites, as confirmed by SEM and a higher double-layer capacitance. Simultaneously, the intimate interfacial contact creates a robust electronic bridge, drastically reducing charge transfer resistance, as evidenced by EIS and facilitating rapid electron transport to the catalytic centres. This harmonious interplay optimizes the adsorption of reaction intermediates and accelerates reaction kinetics, manifesting as exceptionally low overpotentials and small Tafel slopes for both the OER and HER. Ultimately, this synergy between the tailored chemistry of the bimetallic MOF and the superior conductivity of GO forges a durable, high-performance bifunctional electrocatalyst, positioning the NiCu-MOF@GO composite as a frontrunner for next-generation sustainable energy conversion technologies.

## 4 Conclusion

To conclude, the NiCu-MOF@GO composite with superior bifunctional properties for facilitating alkaline water splitting was effectively prepared and tested. The structural characterization verified that the 2D graphene oxide scaffold was very effective in restraining MOF growth, leading to a decrease in the size of the crystallites and very strong chemical interactions (MO–C) between the nodes of the MOF and the carbon matrix. From an electrocatalytic point of view, the composite showed promising performance, with OER and HER overpotentials of 148 mV and 152 mV, respectively, at a current density of 10 mA cm^−2^. The performance was a measurable improvement over that of the pristine MOF and was mechanistically assigned to two reasons: the bimetallic synergy between Ni and Cu, which tunes the electronic structure of the local sites and results in a more optimal intermediate binding energy and the addition of the GO network, which greatly reduces the charge transfer resistance. The HER Tafel slope of 236 mV dec^−1^ suggested that the water dissociation reaction was still a rate-limiting process, but the overall stability and activity of the composite indicated that bimetallic MOFs could be combined with conductive carbon scaffolds to be used in electrochemical energy conversion applications.

## Conflicts of interest

There are no conflicts to declare.

## Data Availability

All relevant data supporting the findings of this study are included in the article. Additional datasets generated and/or analyzed during the current study are available from the corresponding author upon reasonable request.
